# Treatment outcomes and long-term relapse-free survival after multidrug-resistant tuberculosis treatment in Latvia: a retrospective national cohort study

**DOI:** 10.1016/j.lanepe.2026.101676

**Published:** 2026-04-09

**Authors:** Sophie Charlotte Meier, Liga Kukša, Santa Ķauķe, Vija Riekstina, Evita Biraua, Nityanand Jain, Christoph Lange, Thomas Theo Brehm

**Affiliations:** a Faculty of Medicine, Riga Stradiņš University, 16 Dzirciema Street, Riga, Latvia; b Department of Clinical Infectious Diseases, Research Center Borstel, Leibniz Lung Center, Borstel, Germany; c Riga East University Hospital Centre of TB and Lung Diseases, Riga, Latvia; d Statistics Unit, Riga Stradiņš University, 16 Dzirciema Street, Riga, Latvia; e Respiratory Medicine and International Health, University of Lübeck, Germany; f Baylor College of Medicine and Texas Children's Hospital, Global TB Program, Houston, TX, USA; g Institute for Infection Research and Vaccine Development (IIRVD), University Medical Center Hamburg-Eppendorf, Hamburg, Germany; h German Centre for Infection Research (DZIF), Partner Site Hamburg-Lübeck-Borstel-Riems, Germany; i Division of Infectious Diseases, I. Department of Internal Medicine, University Medical Center Hamburg-Eppendorf, Hamburg, Germany

**Keywords:** Rifampicin-resistance, Relapse-free survival, Drug susceptibility testing, Treatment regimens, Treatment duration, Treatment success, Cure, Expert consilium, TBnet, World Health Organization, Europe

## Abstract

**Background:**

Treatment success rates of multidrug-resistant and rifampicin-resistant tuberculosis (MDR/RR-TB) lag behind those for drug-susceptible TB. However, outcome definitions censoring follow-up at treatment completion may underestimate effectiveness and fail to capture relapse-free survival.

**Methods:**

We conducted a retrospective national cohort study including all adults initiating individualized MDR/RR-TB treatment in the Republic of Latvia between 2005 and 2021. Demographic, clinical, and microbiological data were linked to long-term follow-up on relapse and vital status. Treatment outcomes were classified according to World Health Organization (WHO), TBnet (Tuberculosis Network European Trials Group), expert consilium, and long-term outcome definitions. Predictors of cure were assessed using Firth logistic regression. A landmark analysis at 18 months evaluated the association between treatment duration (≤9 months, 10–17 months, and ≥18 months) and relapse-free survival.

**Findings:**

Among 1299 patients (median age 44 years; 74.9% male), cure rates were 4.8% (n = 62) under WHO definitions, 53.1% (n = 690) under TBnet definitions, and 60.8% (n = 790) under the consilium-based classification. Under long-term follow-up outcome definitions, 56.5% (n = 734) achieved cure and 76.9% (n = 999) achieved relapse-free survival. Receiving ≥3 susceptible drugs independently predicted WHO-defined treatment success (adjusted OR 6.53, 95% CI 2.22–31.69; p < 0.001). In the landmark analysis, treatment duration ≤9 months was associated with higher hazard of relapse or death compared with ≥18 months (HR 1.76, 95% CI 1.03–3.00; p = 0.038), whereas outcomes were similar for 10–17 months vs. ≥18 months (HR 0.71, 95% CI 0.42–1.22; p = 0.22).

**Interpretation:**

In this national cohort treated with individualized MDR/RR-TB regimens, long-term relapse-free outcomes substantially exceeded treatment success defined at treatment completion. These findings support relapse-free survival as complement to end-of-treatment metrics.

**Funding:**

This study received no external funding.

**Research in context**

**Evidence before this study**

We searched PubMed for articles published since Jan 1, 2000 without language restrictions on multidrug-resistant and rifampicin-resistant tuberculosis (MDR/RR-TB) outcomes. We used the following search strategy: ((“multidrug-resistant tuberculosis” [All Fields] OR “MDR-TB” [All Fields] OR “rifampicin-resistant tuberculosis” [All Fields] OR “RR-TB” [All Fields]) AND (“treatment outcomes” [All Fields] OR “relapse” [All Fields] OR “cure” [All Fields])). This search identified no nationwide cohort from a high-burden European country that compared World Health Organization (WHO) and TBnet (Tuberculosis Network European Trials Group) frameworks and included long-term, relapse-free outcomes within the same population.

The initial search was conducted on Oct 4, 2022, before study initiation. We repeated the search on Dec 1, 2025, prior to manuscript submission, using the identical strategy; the repeated search identified additional studies but none that systematically compared WHO and TBnet frameworks and long-term follow-up outcomes within the same national cohort.

Most published studies and programmatic cohorts reported only end-of-treatment outcomes. Long-term, relapse-free outcomes remain poorly documented, and definitions vary widely across studies. National-level data from high-burden European settings are particularly scarce. Few cohorts linked programmatic databases with national vital registries to assess relapse-free survival, and we identified no nationwide cohort from a high-burden European country applying multiple complementary outcome frameworks, including long-term relapse-free outcomes, within the same population.

**Added value of this study**

In this nationwide cohort of 1299 adults with MDR/RR-TB treated under routine programmatic conditions in the Republic of Latvia (2005–2021), we systematically linked clinical data with long-term follow-up and vital status. All patients received individualized MDR/RR-TB regimens overseen by a national expert consilium. By applying four complementary frameworks (WHO, TBnet, expert consilium, and long-term follow-up outcomes), we demonstrate a striking discrepancy between end-of-treatment definitions and patient-relevant long-term prognosis: only 4.8% (n = 62) met WHO definitions for cure, whereas 76.9% achieved relapse-free survival during long-term follow up. Using Firth logistic regression, we identify receiving ≥3 susceptible drugs as the key independent predictor of WHO-defined treatment success. In a landmark analysis at 18 months, treatment durations of 10–17 months were associated with relapse-free survival comparable to durations ≥18 months, whereas ≤9 months was associated with poorer relapse-free survival, within individualized, quality-assured MDR/RR-TB care.

**Implications of all the available evidence**

Our findings indicate that reliance solely on end-of-treatment categories substantially underestimates programmatic effectiveness in MDR/RR-TB care. Incorporating long-term, relapse-free endpoints provides a more meaningful reflection of patient benefit and system performance. Ensuring that treatment regimens include at least three susceptible drugs, enabled by rapid and comprehensive drug-susceptibility testing (DST), is central to achieving cure. Within expert-guided individualized care, intermediate treatment durations may achieve relapse-free outcomes comparable to longer courses when regimen potency is maintained while very short treatment durations are associated with worse outcomes. Programmes should prioritize rapid DST, sustained regimen potency, and routine tracking of relapse-free survival. Prospective studies are needed to validate these observations in the era of shorter, bedaquiline-containing treatment regimens for MDR/RR-TB.

## Introduction

Tuberculosis (TB) remains a major global health challenge, with an estimated 10.7 million cases and approximately 1.23 million deaths, making it the leading cause of death from a single infectious agent in 2024.^1^ Multidrug-resistant and rifampicin-resistant TB (MDR/RR-TB) poses a particular challenge, with an estimated 390,000 cases worldwide that year.^2^

Before 2019, World Health Organization (WHO)-recommended individualized regimens for MDR/RR-TB lasted 18–24 months and included injectable agents, which were administered for 4–8 months.^3^ In 2020, the WHO endorsed a 9-month fully oral short treatment regimen for eligible patients with MDR/RR-TB.^4^ More recently, the WHO has further prioritized all-oral, shorter regimens as the preferred standard of care, including 6-month (BPaLM and BDLLfxC) and 9-month (BLMZ, BLLfxCZ, and BDLLfxZ) bedaquiline-containing options.^5^ These regimens have shown higher treatment success rates than historical individualized regimens. Despite these advances, real-world treatment outcomes still vary considerably depending on the resistance profiles. While the treatment success rate for drug-susceptible TB is approximately 88%, it is only around 71% for MDR/RR-TB, 63% for pre-extensively drug-resistant (pre-XDR) TB, and as low as 40% for extensively drug-resistant (XDR) TB based on WHO definitions, which assess treatment success solely at the end of therapy.^1^^,^^6^^,^^7^

We previously demonstrated that among 163 patients treated at the Research Center Borstel in Germany between 2002 and 2020, only 50.3% were cured according to WHO treatment outcome definitions.^8^ However, among those with known follow-up status, 82.8% achieved long-term, relapse-free survival after a median follow-up of four years. These findings highlight the limitations of end-of-treatment outcome measures and suggest that relapse-free survival provides a more meaningful measure of treatment effectiveness. However, comparable data from other European countries with a higher burden of MDR/RR-TB remain scarce.

The Republic of Latvia had an estimated TB incidence of 21 cases per 100,000 population in 2024; 9.1% of new TB cases and 24% of previously treated TB cases presented with MDR/RR-TB.^1^ This places the Republic of Latvia among the high-burden countries for MDR/RR-TB within the European Union/European Economic Area (EU/EEA).^9^

By analysing national MDR/RR-TB data from the Republic of Latvia between 2005 and 2021, we aimed to assess programmatic treatment effectiveness using both conventional and long-term outcome definitions, including relapse-free survival, within a national programme in which all patients were treated with individualized MDR/RR-TB regimens overseen by a national expert consilium. We hypothesized that end-of-treatment outcome definitions substantially underestimate true long-term treatment success in MDR/RR-TB compared with alternative classification frameworks, and that relapse-free survival provides a more patient-relevant measure of programmatic effectiveness.

## Methods

### Study population

This retrospective cohort study included all adult (≥18 years) patients who initiated treatment for MDR/RR-TB in the Republic of Latvia between January 2005 and December 2021. Repeated episodes in the same patient were not considered as separate observations. Patients were excluded if key data required for outcome classification were missing (e.g. treatment start date or assigned treatment outcome). Eligible patients were identified from the National MDR-TB database. All patients initially received inpatient treatment at the Center of Tuberculosis and Lung Diseases (CTLD), where regimens were centrally defined and approved by the national expert consilium; subsequent regional care reflected decentralized treatment delivery. Demographic, clinical, and microbiological data were extracted from the National Tuberculosis Registry through a structured review of patient records conducted between October 4, 2022, and January 31, 2024. Comorbidities were assessed using the Charlson Comorbidity Index (CCI).^10^ Alcohol abuse was not defined using predefined quantitative criteria but was assessed retrospectively based on the treating physicians’ clinical judgement as documented in the medical records and discharge summaries.

Follow-up data, including date and health status at the last medical contact, were obtained through direct communication with outpatient departments or by reviewing local medical records between October 22, 2024, and February 28, 2025. Information on survival status, date of death, and cause of death (if applicable) was provided by the Center for Disease Prevention and Control of the Republic of Latvia as of April 17, 2024. We followed the STROBE guidelines for reporting of observational studies.^11^

### Drug-resistance profiles and treatment regimens

Microbiological diagnostics during the study period included culture and phenotypic drug susceptibility testing (pDST), performed using Mycobacteria Growth Indicator Tubes (MGIT; Becton Dickinson, Franklin Lakes, NJ, USA) or solid Löwenstein-Jensen media. In addition, selected patients underwent genotypic testing using line-probe assays (LPA), including INNO-LiPA® Rif. TB (Innogenetics, Ghent, Belgium) and GenoType® MTBDRplus (Hain Lifescience, Nehren, Germany), available since 2006. Since 2010, Xpert® MTB/RIF (Cepheid, Sunnyvale, CA, USA) has also been in routine use. For evaluation of treatment regimens, the final regimen after availability of DST results was used. Only first-line anti-TB drugs and drugs classified in WHO groups A, B, and C were considered in the analysis of drug-resistance profiles and treatment regimens; other substances that were occasionally used were not included. For analysis, the date of the first microbiological detection of *Mycobacterium tuberculosis* was used rather than the start of treatment, which means that isolates identified in the calendar year 2004 were also included if the corresponding patients initiated treatment between January 2005 and December 2021, in accordance with the study inclusion criteria.

### Treatment outcome according to different definitions

Treatment outcomes were categorized based on the WHO definition of 2021^12^ (Table 1). As all patients in our cohort received individualized treatment regimens, a regimen change was classified as treatment failure only if at least two drugs were permanently replaced due to adverse drug reactions, consistent with considerations raised in the WHO expert consultation on drug-resistant TB treatment outcome definitions. In addition, the TBnet (Tuberculosis Network European Trials Group) outcome definitions were applied.^13^ As MDR/RR-TB treatment regimens in the Republic of Latvia are determined by a national consilium, we additionally used an adapted outcome classification to account for this centralized treatment approach. Patients whose treatment was formally terminated by the consilium before completing the WHO-recommended 18 months were classified as ‘treatment completed’ or ‘cured,’ provided that all other clinical and microbiological criteria were fulfilled. Under standard WHO definitions, these patients would otherwise have been categorized as ‘lost to follow-up.’ Treatment outcome assignment by the consilium was based on case-by-case clinical judgment and was not guided by predefined or standardized criteria. In addition, long-term follow-up was defined at two years after treatment completion. “Cure” was assigned to patients who were alive and relapse-free at censoring. “Completed” referred to patients for whom relapse, death, or cure could not be determined. Death was defined as any fatal event during treatment or within two years of its completion. Relapse required microbiological confirmation, and patients who transferred out during treatment were classified as not evaluated.

**Table 1:** Treatment outcome definitions according to WHO 2021, TBnet, and long-term follow-up.

	WHO 2021^12^	TBnet^13^	Long-term follow-up
Cured	A patient with pulmonary TB with bacteriologically confirmed TB at the beginning of treatment who completed treatment as recommended by the national policy, with evidence of bacteriological response^a^ and no evidence of failure.	A negative culture status 6 months after treatment initiation, no positive cultures thereafter, and no relapses within 1 year after treatment completion	A patient without recurrent TB who is alive at least 2 years after the end of treatment
Treatment completed	A patient who completed treatment as recommended by the national policy but whose outcome does not meet the definition for cure or treatment failure.	N/A	A patient who completed treatment as recommended by the national policy without failure or evidence of active disease at the end of treatment for whom no longtime treatment outcome cure, death or relapse could be assigned because they were lost to-follow-up or transferred out within the first 6 months after the end of treatment.
Treatment failed	A patient whose treatment regimen needed to be terminated or permanently changed to a new regimen or treatment strategy.^b^^,^^c^	A positive culture status 6 month after treatment initiation or thereafter or a relapse within 1 year after treatment completion	N/A
Died	A patient who died before starting treatment or during the course of treatment.^d^	Patient who died for any reason during the course of observation	Patient who died for any reason during the course of observation
Lost to follow-up	A patient who did not start treatment or whose treatment was interrupted for 2 consecutive months or more.	Nonreceipt of care 6 months after treatment initiation	N/A
Not evaluated	A patient for whom no treatment outcome was assigned^e^	N/A	A patient for whom no longtime treatment outcome cure, death or relapse could be assigned because they were lost to-follow-up or transferred out during treatment.
Undeclared outcome	N/A	No culture status at 6 months while the patient was receiving care or no posttreatment assessment	N/A
Relapse	N/A	N/A	A patient who experienced recurrence of tuberculosis after the end of treatment.

DS-TB = drug-susceptible tuberculosis; DR-TB = drug-resistant tuberculosis; N/A = not applicable; TB = Tuberculosis; TBnet = Tuberculosis Network European Trials Group; WHO = World Health Organization.

a “Bacteriological response” refers to bacteriological conversion with no reversion. • “bacteriological conversion” describes a situation in a patient with bacteriologically confirmed TB where at least two consecutive cultures (for DR-TB and DS-TB) or smears (for DS-TB only) taken on different occasions at least 7 days apart are negative; and • “bacteriological reversion” describes a situation where at least two consecutive cultures (for DR-TB and DS-TB) or smears (for DS-TB only) taken on different occasions at least 7 days apart are positive either after the bacteriological conversion or in patients without bacteriological confirmation of TB.

b Reasons for the change include. • no clinical response or no bacteriological response, or both (see note ‘a’); • adverse drug reaction; or • evidence of additional drug-resistance to medicines in the regimen.

c Based on considerations raised in the WHO expert consultation on drug-resistant tuberculosis treatment outcome definitions, when a patient is on a standardized treatment regimen, regimen change implies a change of the whole regimen; in contrast, when a patient is on an individualized treatment regimen, regimen change implies a change of at least two drugs in the regimen.

d Patient died for any reason.

e This includes cases “transferred out” to another treatment unit and whose treatment outcome is unknown; however, it excludes those lost to follow-up.

We used a Sankey diagram to visualize patient trajectories across four classification systems: the WHO classification, the TBnet classification, the outcome of the expert consilium discussion, and the long-term follow-up outcome after treatment. The data were structured to capture transitions between these stages for each patient. Transitions between adjacent layers were counted, and a directed flow diagram was constructed.

### Multivariable logistic regression of factors linked to cure

We performed multivariable Firth penalized logistic regression analyses to identify predictors of successful treatment outcome (cure or treatment completed), defined according to the WHO and the TBnet treatment outcome definitions. Candidate predictors were selected *a priori* based on clinical relevance and data availability: sex, age (categorized at the cohort median), excess alcohol use, human immunodeficiency virus (HIV) status, CCI (0–1 vs. ≥2), and the number of drugs tested as susceptible in the treatment regimen (<3 vs. ≥3). Drugs were considered susceptible if phenotypic or molecular drug susceptibility testing confirmed sensitivity to agents belonging to WHO group A, B, or C. Because of the limited sample size and the potential for data separation, Firth's bias-reduced penalized likelihood logistic regression was used to obtain robust and unbiased estimates. Each predictor was first evaluated in a univariable model, followed by a multivariable model including all candidate predictors. Results are reported as adjusted odds ratios (aORs) with corresponding 95% confidence intervals (CIs) and p-values derived from the profile penalized likelihood.

### Landmark analysis for relapse-free survival according to total treatment duration

To assess the association between treatment duration and relapse-free survival, we performed a landmark analysis with a predefined time point at 18 months after treatment initiation. Patients were included if they had at least 18 months of documented follow-up after the start of treatment. The primary outcome was a composite event defined as either relapse or death occurring after the 18-month landmark. Relapse was defined as a documented recurrence of TB after completion of the initial treatment, with the date of relapse occurring more than 18 months after treatment initiation. Death was considered an event if it occurred more than 18 months after treatment initiation. Observation time was calculated from the 18-month landmark until the last follow-up, with censoring at 24 months after the landmark. This landmark approach was chosen to mitigate immortal time bias when comparing total treatment duration groups. Relapse-free survival was therefore evaluated from the 18-month landmark onward among patients who were alive and relapse-free at that time point. Treatment duration was categorized into three groups: ≤9 months, 10–17 months and ≥18 months. Relapse-free survival was estimated using Kaplan–Meier curves with 95% confidence intervals, and differences between groups were assessed by log-rank test. Cox proportional hazards models were fitted to estimate hazard ratios (HR) and 95% CIs, using treatment duration ≥18 months as the reference category. The proportional hazards assumption was assessed using scaled Schoenfeld residuals and visual inspection of residual plots.

### Statistical analyses

Data were extracted and stored using Microsoft Excel worksheets. Descriptive analyses were done in Microsoft Excel using Pivot tables. All other analyses were done using R (version 4.5.1) in RStudio (version 2025.05.1 + 513) or GraphPad Prism for Mac, version 10.3.1 (San Diego, California, USA).

### Ethics approval

The study protocol was approved by the Ethics Committee of Riga Stradins University (Riga, Latvia; approval No. 2-PĒK-4/333/2023; April 6, 2023) and by the Science Department of Riga East Clinical University Hospital (Riga, Latvia; approval No. AP/08-08/23/112; May 9, 2023). The requirement for informed consent was waived due to the retrospective design of the study and the use of anonymised routinely collected data.

### Role of the funding source

This study received no external funding.

## Results

### Study cohort

**Fig. 1:**
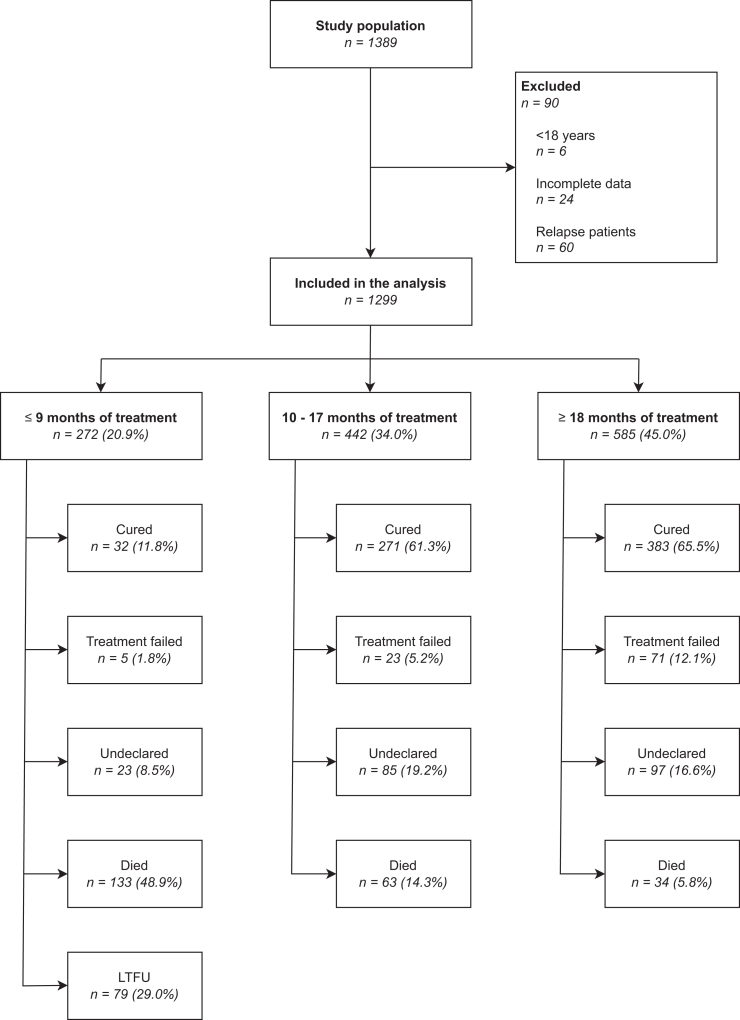
Flowchart of the study population and outcomes according to TBnet (Tuberculosis Network European Trials Group) definitions. **Legend:** Flowchart illustrating the study population and final treatment outcomes stratified by treatment duration, according to TBnet definitions. The mini-bar plots display the distribution of final outcomes at the end of each treatment duration category. Both absolute numbers and percentages are shown. Percentages are calculated as the proportion of patients with a given final outcome relative to the total number of patients who completed treatment within the respective duration category. LTFU = lost to follow-up.

From an initial dataset of 1389 patients registered with MDR/RR-TB during the study period, six patients were excluded because they were younger than 18 years, 60 because they represented recurrent treatment episodes and thus appeared more than once in the database, and 24 because missing key data precluded reliable classification of treatment outcomes (Fig. 1). Given the small proportion of exclusions due to missing outcome-defining data, multiple imputation was not performed. The final cohort constituted 1299 patients. Among these, 973 patients (74.9%) were male, with a median age of 44 years (interquartile range [IQR]: 34–53 years) (Table 2). Most cases (91.7%, n = 1191) presented with pulmonary TB as the predominant site of disease, with the remainder having extrapulmonary disease. Alcohol abuse was reported in 31.6% (n = 411), and a history of intravenous drug use in 9.4% (n = 121). A total of 209 patients (16.1%) had a history of incarceration, and 77 patients (5.9%) were incarcerated at the time of diagnosis. HIV coinfection was documented in 12.1% (n = 157). Most patients had a CCI of 0 (57.8%, n = 751) or 1 (27.1%, n = 352), indicating a relatively low overall comorbidity burden.

**Table 2:** Baseline characteristics of the study cohort.

Characteristics (n = 1299)	n	%
Gender		
Male	973	74.9
Female	326	25.1
Age years, median, IQR	44 (34–53)	
Site of infection		
Pulmonary TB	1191	91.7
Extrapulmonary TB	108	8.3
Drug resistance pattern		
MDR/RR-TB	1069	82.3
Pre-XDR TB	230	17.7
Charlson Comorbidity Index		
0	751	57.8
1	352	27.1
2	134	10.3
3	40	3.1
≥4	22	1.7
HIV infection	157	12.1
Alcohol abuse	411	31.6
TB in prison		
Formerly incarcerated	209	16.1
Currently incarcerated	77	5.9
Prison guard	4	0.31
Year of treatment initiation		
2005	155	11.9
2006	136	10.5
2007	111	8.5
2008	110	8.5
2009	126	9.7
2010	76	5.9
2011	95	7.3
2012	104	8.0
2013	64	4.9
2014	63	4.8
2015	53	4.1
2016	48	3.7
2017	41	3.2
2018	38	2.9
2019	45	3.5
2020	18	1.4
2021	16	1.2

HIV = human immunodeficiency virus; IQR = interquartile range; TB = tuberculosis; MDR/RR = multidrug-resistant or rifampicin-resistant; XDR = extensively drug-resistant.

### Drug-resistance profiles

All *M.* *tuberculosis* isolates had documented resistance to both rifampicin and isoniazid, fulfilling the definition of MDR-TB. A total of 17.6% (n = 228) of isolates demonstrated additional resistance to fluoroquinolones and were therefore classified as having at least pre-XDR-TB (pre-extensively drug-resistant-TB). Linezolid susceptibility testing was not performed in most isolates (81.3%, n = 1056). Among the tested isolates, 18.6% (n = 242) were found to be susceptible, and resistance was detected in only one fluoroquinolone-susceptible isolate. Resistance testing to bedaquiline, clofazimine, and delamanid was introduced in 2017. No resistance to bedaquiline or clofazimine was detected, and resistance to delamanid was observed in fewer than 10% of isolates. For cycloserine/terizidone, resistance was observed in 2.7% (n = 35) of all isolates, susceptibility in 90.2% (n = 1172), and no testing was performed in 7.1% (n = 92). Resistance to pyrazinamide was observed in 69.1% (n = 898) of all isolates, while 26.6% (n = 345) were susceptible and 4.3% (n = 56) were not tested. Ethambutol resistance was present in 78.7% (n = 1022) of all isolates, with 20.6% (n = 268) being susceptible and 0.7% (n = 9) untested. Resistance to amikacin was observed in 26.2% (n = 340) of all isolates, with 41.8% (n = 543) being susceptible and 32.0% (n = 416) not tested. Kanamycin resistance was present in 43.8% (n = 569) of all isolates, susceptibility in 51.2% (n = 665), and 5.0% (n = 65) were not tested. For capreomycin, resistance was observed in 43.0% (n = 559) of all isolates, with 52.4% (n = 681) susceptible and 4.5% (n = 59) untested. PAS resistance was detected in 16.3% (n = 212) of all isolates, while 76.3% (n = 991) were susceptible and 7.4% (n = 96) were not tested.

### Trends in resistance rates

**Fig. 2:**
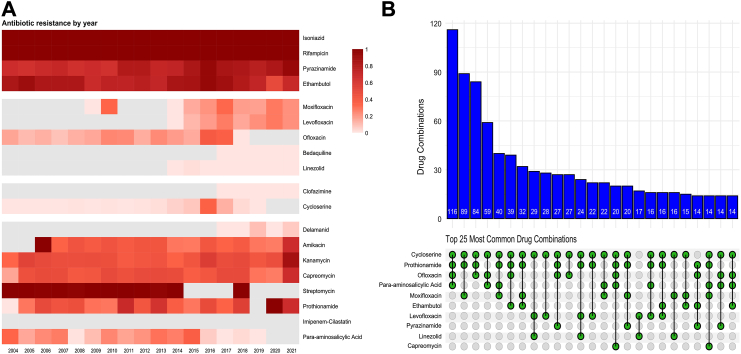
Trends in antibiotic resistance and treatment regimens. **Legend:** (A) Heatmap of antibiogram profiles over the study period. Darker shades represent a higher proportion of resistant isolates, calculated as the ratio of the number of resistant isolates in comparison to the total number of isolates tested for each antibiotic each year. Year corresponds to the year of patient diagnosis, which may differ from the year when resistance testing was performed; (B) Upset plot of the 25 most frequently prescribed treatment regimens, with blue bars showing the frequency of each regimen and dots and lines indicating the respective drug combinations.

Between 2004 and 2021, resistance to pyrazinamide increased substantially (from 66.7% to 92.9%), whereas ethambutol resistance declined (from 88.3% to 69.2%) (Fig. 2A. Among the fluoroquinolones, ofloxacin was the main agent tested until 2013, after which routine testing for levofloxacin and moxifloxacin was introduced. Overall fluoroquinolone resistance remained relatively stable. Cycloserine/terizidone resistance was initially rare but peaked at 34.8% in 2016, followed by a marked decline thereafter. For injectable agents, amikacin resistance was 100% in 2006, fluctuated between 25% and 45% from 2007 to 2020, and rose again to 66.7% in 2021. Kanamycin and capreomycin resistance increased steadily throughout the study period, both exceeding 65% by 2021. Streptomycin resistance remained consistently high across all years. Resistance to para-aminosalicylic acid (PAS) showed a fluctuating pattern, declining from 33.3% in 2004 to 0% in 2008, increasing again to 36.5% in 2015, and ultimately declining to 0% by 2019.

### Treatment regimens

All patients received individualized treatment regimens. The final treatment regimen included a median of four anti-TB drugs (IQR 4–5), with a median of two agents (IQR 1–3) confirmed as susceptible by DST. Drugs included in the treatment regimens comprised first-line anti-TB drugs such as isoniazid (n = 13, 1.0%), rifampicin (n = 8, 0.62%), pyrazinamide (n = 247, 19.0%), and ethambutol (n = 284, 21.9%). Group A drugs included moxifloxacin (n = 415, 31.9%), levofloxacin (n = 260, 20.0%), linezolid (n = 196, 15.1%), and bedaquiline (n = 85, 6.5%). Group B drugs consisted of terizidone or cycloserine (n = 1152, 88.7%) and clofazimine (n = 21, 1.6%). Group C drugs included amikacin (n = 32, 2.5%), delamanid (n = 74, 5.7%), imipenem-cilastatin (n = 8, 0.62%), prothionamide (n = 836, 64.4%), para-aminosalicylic acid (n = 470, 36.2%), and streptomycin (n = 1, 0.08%). The most frequently prescribed treatment regimens were terizidone/cycloserine, prothionamide, ofloxacin, and para-aminosalicylic acid (n = 116), terizidone/cycloserine, prothionamide, and moxifloxacin (n = 89), as well as terizidone/cycloserine, prothionamide, and ofloxacin (n = 84). In total, 250 distinct antibiotic combinations were used, reflecting highly individualized treatment approaches (Fig. 2B).

Median treatment duration was 17.4 months (IQR 10.9–19.9 months). Overall, 20.3% (n = 264) of patients received treatment for less than 9 months, 7.7% (n = 100) between 9 and 12 months, 10.8% (n = 140) between 12 and 15 months, 16.1% (n = 209) between 15 and 18 months and 45.1% (n = 586) completed 18 months or more of treatment. Shortened treatment durations were attributed to death in 8.8% (n = 114), treatment interruption or loss to follow up in 17.2% (n = 224), transferred out/left the cohort in 0.3% (n = 4), or expert consilium decisions in 28.6% (n = 371).

### Treatment outcomes

**Fig. 3:**
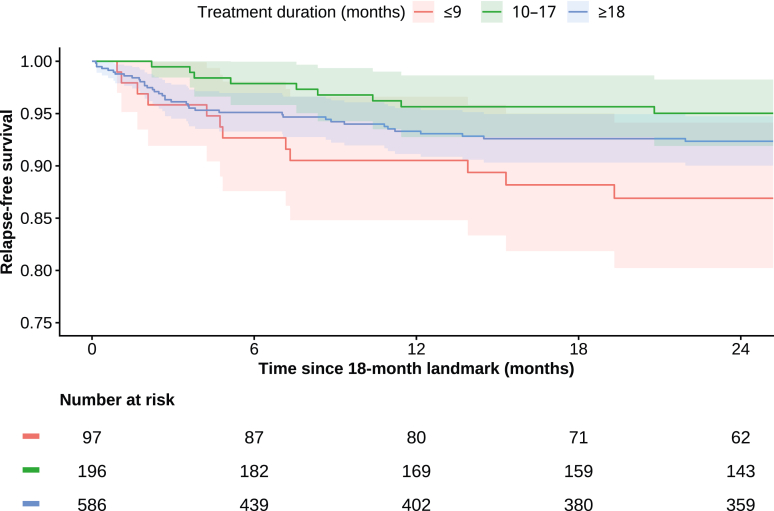
Landmark analysis of relapse-free survival by treatment duration. **Legend:** Kaplan–Meier curves of relapse-free survival starting 18 months after treatment initiation, stratified by total treatment duration (≤9 months, 10–17 months, ≥18 months). Shaded areas indicate 95% confidence intervals. The number of patients at risk at different time points up to 24 months after the 18-month landmark is shown below the plot.

Treatment duration varied substantially, with 20.9% (n = 272) of all patients receiving ≤9 months of treatment, 34.0% (n = 442) 10–17 months, and 45.0% (n = 585) ≥18 months of treatment. Outcomes differed across these treatment duration groups (Fig. 3).

Follow-up was available for at least one year after treatment completion in 65.3% (n = 848) of patients, for at least two years in 57.6% (n = 748), and for at least five years in 39.6% (n = 514). In total, 23.7% (n = 308) of patients had no follow-up after treatment completion. The maximum observed follow-up duration was 19.9 years.

**Fig. 4:**
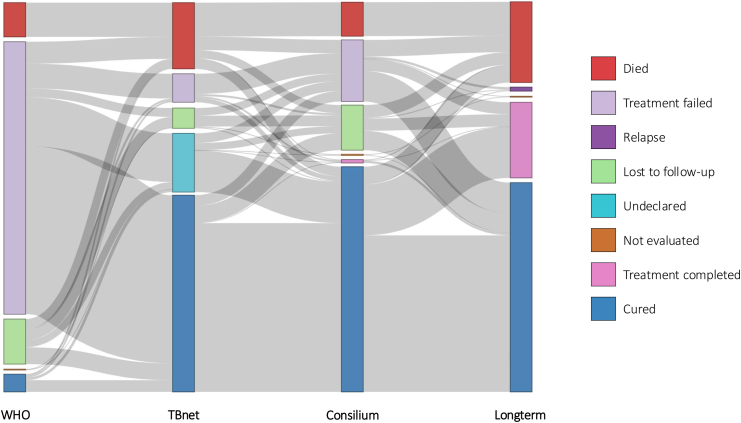
Sankey diagram of treatment outcomes across different definitions. **Legend:** The figure illustrates the flow of treatment outcomes for the study population according to the WHO definitions, TBnet definitions, consilium definitions, and long-term follow-up. Widths of the flows are proportional to the number of patients moving between categories across definitions.

According to the WHO definitions, cure was achieved in 4.8% (n = 62) of patients (Fig. 4). Treatment failure occurred in 73.6% (n = 956), 9.2% (n = 120) died during treatment, 12.2% (n = 158) were lost to follow-up, and 0.2% (n = 3) were not evaluated. Out of all patients that were classified as treatment failures according to WHO definitions, 81.5% were assigned this status because of a change in two or more anti-TB drugs due to adverse events, not due to insufficient clinical or microbiological response. If these treatment regimen changes had not been classified as treatment failure, 64.7% (n = 841) of patients would have met the WHO criteria for cure.

According to TBnet definitions, 53.1% (n = 690) of patients were classified as cured, 7.7% (n = 100) experienced treatment failure, 17.9% (n = 232) died, 5.5% (n = 71) were lost to follow-up, and 15.9% (n = 206) had an undeclared outcome. Successful treatment, defined as cure or treatment completion, was achieved in 53.1% (n = 690) of patients.

According to the national consilium's decision, 60.8% (n = 790) were considered cured, 0.9% (n = 12) completed treatment, 16.6% (n = 216) experienced treatment failure, 9.2% (n = 119) died, 12.2% (n = 158) were lost to follow-up, and 0.3% (n = 4) were not evaluated. Successful treatment was achieved in 61.7% (n = 802) of patients.

Based on the long-term outcome criteria, 56.5% (n = 734) were cured, 20.4% (n = 265) completed treatment, 21.8% (n = 283) died, 1.1% (n = 14) had a relapse, and 0.2% (n = 3) were not evaluated. Successful treatment was achieved in 76.9% (n = 999) of patients.

### Multivariable analyses of treatment outcomes

**Fig. 5:**
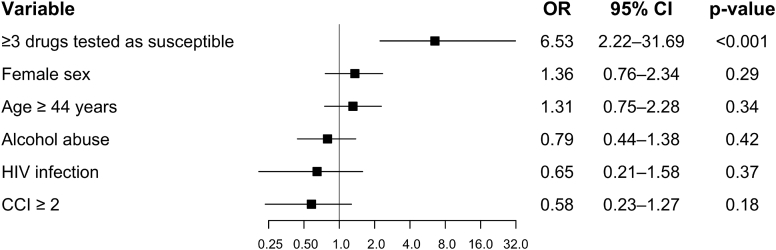
Factors associated with cure in patients with multidrug-resistant tuberculosis. **Legend:** Forest plot showing adjusted odds ratios (OR) and 95% confidence intervals (95% CI) for variables associated with cure defined by WHO outcomes. Estimates were obtained using multivariable Firth logistic regression to account for potential small-sample bias. The model was adjusted for number of drugs tested as susceptible (≥3), female sex, age (≥median age 44 years), alcohol abuse, Charlson Comorbidity Index (CCI ≥2), and human immunodeficiency virus (HIV) infection.

In the multivariable Firth penalized logistic regression analysis using the WHO definition of successful treatment outcome, the number of susceptible drugs in the treatment regimen was the only factor independently associated with treatment success (Fig. 5). Patients receiving ≥3 drugs tested as susceptible had markedly higher odds of a successful treatment outcome compared to those receiving fewer than three susceptible drugs (aOR 6.53 (95% CI 2.22–31.69)). None of the other variables, including female sex (aOR 1.36, 95% CI 0.76–2.34), age ≥44 years, dichotomized at the cohort median (aOR 1.31, 95% CI 0.75–2.28), excess alcohol use (aOR 0.79, 95% CI 0.44–1.38), HIV infection (aOR 0.65, 95% CI 0.21–1.58), or a CCI ≥2 (aOR 0.58, 95% CI 0.23–1.27)–were significantly associated with treatment success. The overall model likelihood ratio test was statistically significant (χ^2^ = 19.76, df = 6, p = 0.003).

In an additional analysis using TBnet-defined successful treatment outcome, female sex was associated with higher odds of treatment success (aOR 1.79, 95% CI 1.32–2.43). In contrast, HIV infection (aOR 0.27, 95% CI 0.18–0.41), excess alcohol use (aOR 0.72, 95% CI 0.55–0.94), and a CCI ≥2 (aOR 0.58, 95% CI 0.38–0.88) were associated with significantly reduced odds of treatment success. Age ≥44 years was not significantly associated with the outcome. In contrast to the WHO outcome-based analysis, receipt of ≥3 susceptible drugs was also associated with lower odds of treatment success (aOR 0.65, 95% CI 0.47–0.90). The overall model likelihood ratio test was statistically significant (χ^2^ = 75.11, df = 6, p < 0.001).

### Landmark analysis for relapse-free survival according to total treatment duration

A total of 948 patients were eligible for the landmark analysis; 879 patients with complete data were included in the Cox regression, contributing 96 events. Median follow-up from the 18-month landmark was 24.0 months (IQR 12.9–24.0) when administratively censored at 24 months; uncensored follow-up was 49.1 months (IQR 12.9–108.4). Relapse-free survival differed across treatment-duration groups (log-rank p = 0.02). In Cox proportional hazards regression with ≥18 months as the reference category, treatment durations of ≤9 months were associated with a significantly higher hazard of relapse or death (HR 1.76, 95% CI 1.03–3.00, p = 0.038). Treatment durations of 10–17 months were not significantly associated with relapse-free survival (HR 0.71, 95% CI 0.42–1.22, p = 0.22). There was no statistically significant evidence of violation of the proportional hazards assumption (global test p = 0.056).

## Discussion

In this nationwide cohort of 1299 adults with MDR/RR-TB treated under programmatic conditions in the Republic of Latvia, we report long-term outcomes that substantially exceed those captured by conventional WHO end-of-treatment definitions.^14^ While only 4.8% of patients met WHO criteria for cure, 76.9% were alive and relapse-free on long-term follow-up.^15^^,^^16^ These findings, representing one of the largest national analyses to date linking routine programmatic data with long-term outcomes in a high-burden European setting, highlight the limitations of relying solely on end-of-treatment outcome categories in MDR/RR-TB.

When placed in the context of treatment success reported across different regimens, our results are consistent with broader programmatic experience. In a recent systematic review and meta-analysis covering 2009–2024, the pooled treatment success for MDR/RR-TB in observational studies was 68.4%, with a gradual increase in success rates over time.^17^ This improvement has been most pronounced with the introduction of newer all-oral, bedaquiline-containing regimens, which were not yet implemented in the Republic of Latvia during the study period. Across clinical trials and programmatic studies, 6–9-month bedaquiline-containing regimens have achieved favourable outcomes in approximately 80–93% of patients: 6-month BPaL(M) regimens in Nix-TB,^18^ ZeNix,^19^ and TB-PRACTECAL (84–93%)^20^; different 9-month regimens (BCLLfxZ, BLMZ, and BDLLfxZ) in endTB (∼80%)^21^; the 6-month BDLLfxC regimen in BEAT Tuberculosis (86%)^22^; and the 6–9-month BDLC regimen in endTB-Q (87%).^23^ These findings are further supported by real-world programmatic evidence from the WHO European Region^24^, ^25^, ^26^, ^27^ and other settings,^28^, ^29^, ^30^ demonstrating similarly high treatment success under routine care conditions.

Beyond treatment success at treatment completion, an increasing body of evidence highlights the importance of extended follow-up to capture patient-relevant outcomes. We previously demonstrated that among 163 patients treated at the Research Center Borstel in Germany between 2002 and 2020, only 50.3% were cured according to WHO treatment outcome definitions; however, among those with known follow-up status, 82.8% achieved long-term, relapse-free survival after a median follow-up of four years. In a previous cohort study spanning 16 European countries, WHO-defined cure was only 39% in high-incidence settings and 10% in low-incidence settings, whereas TBnet outcome definitions incorporating one year of post-treatment follow-up yielded similar relapse-free survival rates of approximately 56–58%.^31^ More recently, multicountry programmatic data from the WHO European Region evaluating fully oral modified short treatment regimens reported a cumulative probability of 79% for having a successful outcome up to 22 months after treatment initiation,^32^ a figure closely aligned with the treatment success observed in our cohort.

A central explanation for the discrepancy between WHO outcomes and long-term prognosis is definitional: in our cohort, 73.6% fulfilled the WHO criterion failure, which in 81.5% was driven by adverse events prompting regimen modification rather than by microbiological or clinical non-response. Individualized regimens requiring the replacement of two or more drugs due to adverse events are classified as treatment failure.^33^ We retrospectively assessed regimen modifications from routine clinical documentation, without systematic information on adverse event type or severity. This may have resulted in conservative classification of toxicity-related regimen changes as treatment failure and may partly explain why the proportion of patients with successful treatment outcomes under WHO definitions was lower in our study than in comparable European cohorts. Notably, if regimen changes were not classified as treatment failure, 64.7% of patients would have met the WHO criteria for cure. Taken together, our findings suggest that end-of-treatment categories may underestimate long-term relapse-free survival in settings where individualized regimens are frequently adapted for tolerability and safety.

Treatment duration emerged as an important correlate of prognosis in the landmark analysis. Using ≥18 months as the reference category, treatment durations of <9 months were associated with a significantly higher risk of relapse or death, whereas outcomes among patients treated for 10–17 months were comparable to those treated for ≥18 months. These data suggest that, within individualized care pathways overseen by a national expert consilium, treatment courses of intermediate duration may achieve relapse-free survival comparable to longer treatment, provided that regimen quality and drug activity are maintained.

Consistent with this, the number of drugs that were tested susceptible was the only independent predictor of cure by WHO criteria: receiving ≥3 active agents conferred nearly a sixfold higher odds of cure. This finding underscores the programmatic value of rapid, reliable phenotypic and molecular susceptibility testing and highlights the potential of targeted next-generation sequencing (tNGS) as a comprehensive tool for timely detection of resistance.^34^, ^35^, ^36^ Investments into DST scale-up, quality assurance, and especially the integration of tNGS into routine practice are crucial to ensure that patients receive regimens with a sufficient number of susceptible drugs.^37^ In settings transitioning to newer regimens, building and maintaining capacity to determine drug susceptibility–particularly for core drugs–remains essential to preserve regimen effectiveness and prevent the amplification of resistance.^37^, ^38^, ^39^ Notably, associations differed when treatment success was defined using TBnet rather than WHO criteria. Under TBnet definitions, patient-related factors were more strongly associated with treatment success, whereas regimen composition showed an inverse association, with receipt of at least three susceptible drugs being linked to poorer outcomes. This may reflect indication and survivorship bias, as patients with more severe disease, intolerance, or limited treatment options were more likely to receive fewer active drugs and to experience adverse clinical trajectories, rather than a causal effect of regimen composition per se. These findings underscore that WHO and TBnet outcomes capture different dimensions of treatment success.

Our longitudinal resistance analysis showed increasing resistance to pyrazinamide and prothionamide, and to the second-line injectables amikacin, kanamycin, and capreomycin, whereas fluoroquinolone resistance did not increase over time. This pattern differs from several European cohorts where rising fluoroquinolone resistance has been more prominent, and may reflect earlier stewardship of fluoroquinolones in the Republic of Latvia, differential drug exposure histories, or cohort-era effects.^40^ The observed rise in resistance to legacy injectables–now largely deprioritized–highlights the urgency of phasing out toxic, less effective agents and ensuring access to active, all-oral backbones, paired with DST to protect bedaquiline- and linezolid-containing regimens.

Strengths of this study include its national scope, large sample size, and unique linkage of clinical records with vital registry data over nearly two decades, enabling robust ascertainment of relapse and survival. The structured case review by a national consilium provides an informative real-world counterpoint to rigid duration-based paradigms, illustrating how individualized decisions can yield durable outcomes.^41^, ^42^, ^43^, ^44^

### Several limitations merit consideration

First, WHO treatment outcome definitions were operationalized retrospectively using routine clinical documentation. WHO definitions classify permanent regimen change due to adverse events as treatment failure. The operational definition of regimen change applied in this study was based on considerations raised in the WHO expert consultation on drug-resistant tuberculosis treatment outcome definitions, whereby a regimen change in individualized treatment was defined as the permanent replacement of at least two drugs. However, detailed and systematic information on the type and severity of adverse events was not consistently available in our cohort. Consequently, some regimen modifications may have been conservatively categorized as failure, potentially inflating failure rates. In addition, treatment decisions were made within multidisciplinary consilium structures reflecting individualized, patient-centred clinical judgment, which may introduce heterogeneity in thresholds for regimen modification. Our analysis highlights the challenges of applying regimen-based end-of-treatment criteria to retrospective data from individualized care settings. This underscores the value of extended follow-up and relapse-free survival as complementary, patient-relevant measures of long-term effectiveness.

Second, comorbidity burden was assessed using the CCI, which is widely used and validated but may not fully capture multimorbidity compared with more recent scores, potentially resulting in residual confounding.

Third, treatment regimens and durations were heterogeneous, reflecting individualized care, and may have introduced confounding by indication. Generalizability to current practice is further limited by restricted drug susceptibility testing for linezolid and the absence of programmatic DST for bedaquiline and clofazimine during much of the study period.

Fourth, although follow-up was extensive, nearly one quarter of patients lacked post-treatment follow-up, which may have biased long-term estimates if missingness was non-random. In addition, evolving diagnostics and programmatic practices over time may have influenced resistance detection and treatment decisions.

Fifth, the retrospective design did not capture patient-reported outcomes or psychosocial aspects, such as stigma, reintegration, or the individual burden associated with prolonged monitoring. Accordingly, our long-term outcome analyses should be interpreted as an assessment of programmatic effectiveness rather than as an endorsement of intensified post-treatment surveillance at the individual patient level.

Finally, treatment decisions including treatment termination, declaration of cure, or classification of treatment completion were made by a national expert consilium on a case-by-case basis without predefined criteria. While this pragmatic approach reflects real-world practice, it limits reproducibility and external validity.

### Conclusion

Long-term relapse-free cure of MDR/RR-TB is achievable in most patients under programmatic conditions, despite low cure rates by standard criteria. Long-term relapse-free survival exceeded end-of-treatment success in this cohort, but comparisons across outcome frameworks should be interpreted cautiously given potential outcome misclassification and incomplete documentation of adverse event severity and management. Prospective studies incorporating standardized adverse event recording, patient-centred outcomes, and long-term follow-up are needed to validate these observations in the era of shorter, bedaquiline-containing regimens.

## Contributors

S.C.M., L.K., C.L., and T.T.B. developed the concept of the study. S.C.M., V.R., E.B., and S.K. retrieved the data. S.C.M. communicated with the regional TB offices, the Centre for Disease Prevention and Control, and regional health care facilities. S.C.M. curated the data. T.T.B. performed the analyses. S.C.M., L.K., C.L., and T.T.B. interpreted the results. N.J. and T.T.B. were involved in data cleaning, analysis, and visualization. C.L. and L.K. supervised the work. S.C.M. and T.T.B. drafted the manuscript. S.C.M. and T.T.B. had full access to the data and verified the underlying data. S.C.M., C.L., and T.T.B. were responsible for the decision to submit the manuscript. All authors reviewed and approved the final version.

## Data sharing statement

De-identified individual participant data that underlie the results reported in this Article may be made available upon reasonable request to the corresponding author, subject to approval by the relevant institutional and ethical authorities and in accordance with applicable data protection regulations.

## Declaration of interests

C.L. reports personal fees from Astra Zeneca, Gilead, GSK, INSMED, MedUpdate, MedUpdateEurope and Pfizer for lecturing at sponsored symposia outside of the presented work. S.C.M., L.K., S.K., V.R., E.B., N.J. and T.T.B have no interests to declare.

## Appendix A Supplementary data

Translated abstract

## References

[1] (2025). Global Tuberculosis Report 2025.

[2] Organization WH (2024).

[3] WHO (2016). WHO Treatment Guidelines for Drug-Resistant Tuberculosis.

[4] World Health Organization (2020).

[5] World Health Organization (2025).

[6] Pedersen O.S., Holmgaard F.B., Mikkelsen M.K.D. (2023). Global treatment outcomes of extensively drug-resistant tuberculosis in adults: a systematic review and meta-analysis. J Infect.

[7] Kherabi Y., Skouvig Pedersen O., Lange C. (2025). Treatment outcomes of extensively drug-resistant tuberculosis in Europe: a retrospective cohort study. Lancet Reg Health Eur.

[8] Maier C., Chesov D., Schaub D. (2023). Long-term treatment outcomes in patients with multidrug-resistant tuberculosis. Clin Microbiol Infect.

[9] European Center for Disease Prevention and Control (ECDC) and the World Health Organization (WHO) Regional Office for Europe (2024). Tuberculosis Surveillance and Monitoring in Europe 2024 - 2022 Data.

[10] Charlson M.E., Pompei P., Ales K.L., MacKenzie C.R. (1987). A new method of classifying prognostic comorbidity in longitudinal studies: development and validation. J Chronic Dis.

[11] Gallo V., Egger M., McCormack V. (2011). STrengthening the Reporting of OBservational studies in Epidemiology--Molecular Epidemiology (STROBE-ME): an extension of the STROBE Statement. PLoS Med.

[12] World Health Organization Meeting report of the WHO expert consultation on drug-resistant tuberculosis treatment outcome definitions. https://iris.who.int/bitstream/handle/10665/340284/9789240022195-eng.pdf?sequence=1.

[13] Günther G., Lange C., Alexandru S. (2016). Treatment outcomes in multidrug-resistant tuberculosis. N Engl J Med.

[14] (2024). Global Tuberculosis Report 2024.

[15] Jeronimo C.M.P., Farias M.E.L., Val F.F.A. (2021). Methylprednisolone as adjunctive therapy for patients hospitalized with coronavirus disease 2019 (COVID-19; Metcovid): a randomized, double-blind, phase IIb, placebo-controlled trial. Clin Infect Dis.

[16] World Health Organization Definitions and reporting framework for tuberculosis – 2013 revision: updated December 2014 and January 2020. https://iris.who.int/bitstream/handle/10665/79199/9789241505345_eng.pdf?sequence=1.

[17] Nasiri M.J., Amiri M., Cheraghi M. (2025). 15-year trends in efficacy and effectiveness of treatment outcomes in drug-resistant pulmonary TB. IJTLD Open.

[18] Conradie F., Diacon A.H., Ngubane N. (2020). Treatment of highly drug-resistant pulmonary tuberculosis. N Engl J Med.

[19] Conradie F., Bagdasaryan T.R., Borisov S. (2022). Bedaquiline-pretomanid-linezolid regimens for drug-resistant tuberculosis. N Engl J Med.

[20] Nyang'wa B.T., Berry C., Kazounis E. (2024). Short oral regimens for pulmonary rifampicin-resistant tuberculosis (TB-PRACTECAL): an open-label, randomised, controlled, phase 2B-3, multi-arm, multicentre, non-inferiority trial. Lancet Respir Med.

[21] Guglielmetti L., Khan U., Velásquez G.E. (2025). Oral regimens for rifampin-resistant, fluoroquinolone-susceptible tuberculosis. N Engl J Med.

[22] Conradie F., Badat T., Poswa A. (2025). BEAT tuberculosis: a randomized controlled trial of a 6-month strategy for rifampicin-resistant tuberculosis. medRxiv.

[23] Guglielmetti L., Khan U., Velásquez G.E. (2025). Bedaquiline, delamanid, linezolid, and clofazimine for rifampicin-resistant and fluoroquinolone-resistant tuberculosis (endTB-Q): an open-label, multicentre, stratified, non-inferiority, randomised, controlled, phase 3 trial. Lancet Respir Med.

[24] Sinha A., Klebe R., Rekart M.L. (2025). The effectiveness and safety of bedaquiline, pretomanid, and linezolid (BPaL)-Based regimens for rifampicin-resistant tuberculosis in non-trial settings-A prospective cohort study in Belarus and Uzbekistan. Clin Infect Dis.

[25] Gualano G., Musso M., Mencarini P. (2024). Safety and effectiveness of BPaL-Based regimens to treat multidrug-resistant TB: first experience of an Italian tuberculosis referral hospital. Antibiotics (Basel).

[26] Wares D.F., Mbenga M., Mirtskhulava V. (2024). Introducing BPaL: experiences from countries supported under the LIFT-TB project. PLoS One.

[27] Dahl V.N., Skouvig Pedersen O., Butova T. (2025). Effectiveness of the bedaquiline, pretomanid, and linezolid regimen, with or without moxifloxacin, for drug-resistant tuberculosis in Ukraine under programmatic conditions. Clin Infect Dis.

[28] Daniel B.D., Shanmugam S., Mehta R. (2025). Long term outcomes in drug resistant tuberculosis with Bedaquiline, Pretomanid and varying doses of Linezolid. J Infect.

[29] Flores I., Quelapio M.I., Cabalitan C. (2025). Effectiveness and safety of the BPaL regimen in the Philippines. J Clin Tuberc Other Mycobact Dis.

[30] Sangsayunh P., Sanchat T., Chuchottaworn C., Cheewakul K., Rattanawai S. (2024). The use of BPaL containing regimen in the MDR/PreXDR TB treatments in Thailand. J Clin Tuberc Other Mycobact Dis.

[31] Günther G., van Leth F., Alexandru S. (2018). Clinical management of multidrug-resistant tuberculosis in 16 European countries. Am J Respir Crit Care Med.

[32] Korotych O., Achar J., Gurbanova E. (2024). Effectiveness and safety of modified fully oral 9-month treatment regimens for rifampicin-resistant tuberculosis: a prospective cohort study. Lancet Infect Dis.

[33] (2020). Meeting Report of the WHO Expert Consultation on the Definition of Extensively Drug-Resistant Tuberculosis.

[34] Schwab T.C., Perrig L., Göller P.C. (2024). Targeted next-generation sequencing to diagnose drug-resistant tuberculosis: a systematic review and meta-analysis. Lancet Infect Dis.

[35] https://www.who.int/news/item/25-07-2023-who-issues-rapid-communication-on-use-of-targeted-next-generation-sequencing-for-diagnosis-of-drug-resistant-tuberculosis.

[36] Domínguez J., Boeree M.J., Cambau E. (2023). Clinical implications of molecular drug resistance testing for Mycobacterium tuberculosis: a 2023 TBnet/RESIST-TB consensus statement. Lancet Infect Dis.

[37] Chesov E., Chesov D., Maurer F.P. (2022). Emergence of bedaquiline resistance in a high tuberculosis burden country. Eur Respir J.

[38] Günther G., Mhuulu L., Diergaardt A. (2024). Bedaquiline resistance after effective treatment of multidrug-resistant tuberculosis, Namibia. Emerg Infect Dis.

[39] Derendinger B., Dippenaar A., de Vos M. (2023). Bedaquiline resistance in patients with drug-resistant tuberculosis in Cape Town, South Africa: a retrospective longitudinal cohort study. Lancet Microbe.

[40] Konstantynovska O., Synenko T., Honcharenko A. (2025). Fluoroquinolone resistance in drug-resistant tuberculosis, Kharkiv, Ukraine, 2019-2023. Emerg Infect Dis.

[41] Baluku J.B., Katuramu R., Naloka J., Kizito E., Nabwana M., Bongomin F. (2021). Multidisciplinary management of difficult-to-treat drug resistant tuberculosis: a review of cases presented to the national consilium in Uganda. BMC Pulm Med.

[42] Tiberi S., Pontali E., Tadolini M., D'Ambrosio L., Migliori G.B. (2019). Challenging MDR-TB clinical problems–the case for a new Global TB Consilium supporting the compassionate use of new anti-TB drugs. Int J Infect Dis.

[43] Guglielmetti L., Jaffré J., Bernard C. (2019). Multidisciplinary advisory teams to manage multidrug-resistant tuberculosis: the example of the French Consilium. Int J Tuberc Lung Dis.

[44] D'Ambrosio L., Bothamley G., Caminero Luna J.A. (2018). Team approach to manage difficult-to-treat TB cases: experiences in Europe and beyond. Pulmonology.

