# Supplementary material for: Treatment outcomes and long-term relapse-free survival after multidrug-resistant tuberculosis treatment in Latvia: a retrospective national cohort study
Source: Lancet Reg Health Eur. 2026 Apr 9;65:101676. doi: 10.1016/j.lanepe.2026.101676 (PMC13091304; doi:10.1016/j.lanepe.2026.101676)
Supplement: Translated abstract [file mmc1.docx]

This translation in Latvian was submitted by the authors and we reproduce it as supplied. It has not been peer reviewed. Our editorial processes have only been applied to the original abstract in English, which should serve as reference for this manuscript.

**Ārstēšanas rezultāti un ilgtermiņa recidīva brīvā izdzīvotība pēc daudzzāļu rezistentas tuberkulozes ārstēšanas Latvijā: retrospektīvs nacionāls kohortas pētījums**

**Ievads:**
Daudzzāļu vai rifampicīna rezistentas tuberkulozes (MDR/RR-TB) ārstēšanas rezultāti joprojām ir sliktāki nekā zāļu jutīgas tuberkulozes gadījumā. Tomēr ārstēšanas rezultātu definīcijas, kas novērošanu ierobežo līdz ar ārstēšanas pabeigšanu, var novērtēt ārstēšanas efektivitāti par zemu un neatspoguļot izdzīvošanu bez recidīva.

**Metodes:**
Mēs veicām retrospektīvu nacionāla līmeņa kohortas pētījumu, kurā iekļāvām visus pieaugušos pacientus, kuri Latvijā laikā no 2005. līdz 2021. gadam uzsāka individualizētu MDR/RR-TB ārstēšanu. Demogrāfiskie, klīniskie un mikrobioloģiskie dati tika sasaistīti ar ilgtermiņa novērojuma datiem par recidīvu un izdzīvošanas statusu. Ārstēšanas rezultāti tika klasificēti saskaņā ar PVO, TBnet, ekspertu konsīlija un ilgtermiņa iznākumu definīcijām. Izārstēšanas prognozējošie faktori tika novērtēti, izmantojot Firth loģistisko regresiju. Landmark analīzē 18 mēnešus pēc ārstēšanas uzsākšanas tika izvērtēta saistība starp ārstēšanas ilgumu (≤9 mēneši, 10–17 mēneši un ≥18 mēneši) un izdzīvošanu bez recidīva.

**Rezultāti:**
No 1299 pacientiem (mediānais vecums 44 gadi; 74,9% vīrieši) izārstēšanas rādītāji bija 4,8% pēc PVO definīcijām, 53,1% pēc TBnet definīcijām un 60,8% pēc konsīlija balstītas klasifikācijas. Saskaņā ar ilgtermiņa iznākumu definīcijām 56,5% pacientu tika uzskatīti par izārstētiem, bet 76,9% pacientu nebija novērots recidīvs ilgtermiņa novērošanas laikā.Vismaz trīs jutīgu zāļu lietošana ārstēšanas režīmā bija neatkarīgs PVO definēta ārstēšanas panākuma prognozētājs (koriģētais OR 6,53; 95% TI 2,22–31,69; p<0,001). Landmark analīzē ārstēšanas ilgums ≤9 mēneši bija saistīts ar lielāku recidīva vai nāves risku salīdzinājumā ar ≥18 mēnešiem (HR 1,76; 95% TI 1,03–3,00; p=0,038), savukārt rezultāti bija līdzīgi pacientiem ar ārstēšanas ilgumu 10–17 mēneši un ≥18 mēneši (HR 0,71; 95% TI 0,42–1,22; p=0,22).

**Interpretācija:**
Šajā nacionālajā kohortā pacientiem, kuri tika ārstēti ar individualizētiem MDR/RR-TB ārstēšanas režīmiem, ilgtermiņa rezultāti bija būtiski labāki nekā ārstēšanas panākumi, kas noteikti ārstēšanas beigās. Šie rezultāti liecina, ka pacientu ilgtermiņa novērošana pēc ārstēšanas pabeigšanas var būt nozīmīgs papildinājums ārstēšanas rezultātu novērtēšanai.
